# Supplementary material for: WordCommentsAnalyzer: A windows software tool for qualitative research
Source: F1000Res. 2018 Sep 4;7:536. Originally published 2018 May 3. [Version 2] doi: 10.12688/f1000research.14819.2 (PMC6137411; doi:10.12688/f1000research.14819.2)
Supplement: Supplementary file 3 [file f1000research-7-17614-s0002.tgz › b2a79496-4ada-47c4-9682-a99ce867836d.zip!/Eight Iranian Health Journals/Salmand_Iranian Journal of Ageing _13.docx]

© Salmand_Iranian Journal of Ageing . This work is licensed under CC BY-NC 4.0 (https://creativecommons.org/licenses/by-nc/4.0/).

Volume & Issue: دوره 10 - شماره 1

Title: نقش ناتوانی در بخشودگی خود، دیگران و موقعیت‏های کنترل‏ناپذیر در پیش‌بینی افسردگی سالمندان

http://salmandj.uswr.ac.ir/article-1-715-fa.html

اهداف سالمندان رنجش‏های زیادی را تجربه می‌کنند که انتظار می‌رود ناتوانی در بخشش عوامل به وجودآورنده‌ این رنجش‏ها با علائم افسردگی در آنان مرتبط باشد. از این رو پژوهش حاضر با هدف بررسی رابطه بین بخشودگی خود، دیگران و موقعیت‏های کنترل‌ناپذیر با افسردگی در سالمندان انجام گرفت. مواد و روش ها این پژوهش یک مطالعه مقطعی از نوع همبستگی است. جامعه آماری آن 96‌ سالمند زن و مرد عضو کانون‏های جهاندیدگان شهر تهران بودند که به روش نمونه‏ گیری در دسترس گزینش شدند. ابزارهای گردآوری داده‌ها مقیاس بخشودگی هارتلند (HFS) و مقیاس افسردگی سالمندان (GDS) بودند و تجزیه و تحلیل داده‌ها با استفاده از ضریب همبستگی پیرسون و رگرسیون چند متغیره به روش گام به گام انجام شد. یافته ها نتایج نشان داد که بین بخشودگی خود (32/0-=r، 01/0≥P)، بخشودگی دیگران (25/0-=r، 05/0≥P)، بخشودگی موقعیت‏های غیرقابل‌کنترل (38/0-=r، 01/0≥P) با افسردگی سالمندان رابطه منفی معنی‌داری وجود دارد. همچنین دو متغیر بخشودگی موقعیت‏های غیرقابل‌کنترل (33/0-=β) و بخشودگی خود (26/0-=β) به ترتیب نقش تعیین‌کننده‌ای در تبیین متغیر ملاک افسردگی داشتند (01/0>P). نتیجه گیری ناتوانی در بخشش با آسیب‌پذیری روانی سالمندان همبسته است و پیشنهاد می‏شود که مدل‏های درمانی ارتقای بخشش جهت مشکلات هیجانی سالمندان مورد توجه قرار گیرند.

Title: روایی و پایایی آزمون‌های برخاستن و راه‌رفتن زمان‌دار و دسترسی عملکردی قدامی برای ارزیابی خطر زمین‌خوردن در سالمندان

http://salmandj.uswr.ac.ir/article-1-720-fa.html

اهداف هدف پژوهش حاضر بررسی روایی و پایایی آزمون‌های برخاستن و راه‌رفتن زمان‌دار و دسترسی عملکردی قدامی برای ارزیابی خطر زمین‌خوردن در سالمندان بود.  مواد و روش ها بدین منظور 200 آزمودنی سالمند با دامنه سنی 60 سال به بالا، در دو گروه با و بدون سابقه زمین‌خوردن (هر کدام 100 آزمودنی)، انتخاب شدند. همه آزمودنی‌ها آیتم‌های موجود در آزمون‌های برخاستن و راه‌رفتن زمان‌دار را انجام دادند و اطلاعات آن‌ها توسط محقق جمع‌آوری شد. داده‌ها با استفاده از ضریب همبستگی پیرسون، تحلیل واریانس یکراهه، ‌ضریب آلفای کرونباخ و آزمون تی مستقل ارزیابی شدند.  یافته ها آزمون برخاستن و راه‌رفتن زمان‌دار از اعتبار سازه (تفاوت گروهی، روایی بالینی و رشدی)، آلفای کرونباخ (81/0) و ثبات زمانی (98/0) قابل قبولی برخوردار بود. آزمون دسترسی عملکردی نیز از اعتبار سازه (تفاوت گروهی، روایی بالینی و رشدی)، ثبات زمانی (97/0) قابل قبول برخوردار بود. همچنین نتایج تحقیق نشان داد که بین آزمون‌های برخاستن و راه‌رفتن زمان‌دار، دسترسی عملکردی و آزمون برگ همبستگی معنی‌داری وجود دارد.  نتیجه گیری نتایج تحقیق بیانگر روایی و پایایی قابل‌قبول دو آزمون برخاستن و راه‌رفتن زمان‌دار و دسترسی عملکردی در سالمندان بود. لذا از این دو آزمون می‌توان به منظور شناسایی خطر زمین‌خوردن در سالمندان استفاده کرد.

Title: تاثیر یک دوره تمرین ترکیبی هوازی و تعادلی بر تعادل زنان سالمند

http://salmandj.uswr.ac.ir/article-1-743-fa.html

اهداف تعادل، شاخصی برای تعیین میزان استقلال سالمندان در انجام فعالیت‌های روزمره محسوب می‌شود. هدف اصلی از انجام این پژوهش، بررسی تاثیر یک دوره تمرین ترکیبی هوازی و تعادلی بر تعادل ایستا، پویا و عملکردی زنان سالمند بود.  مواد و روش ها این تحقیق از نوع نیمه‌تجربی با طرح پیش آزمون-پس‌آزمون با یک گروه مداخله تمرینی و یک گروه شاهد بود. به همین منظور 30 زن که همگی از سلامت عمومی برخوردار بودند به طور داوطلبانه در این تحقیق شرکت کردند و به روش تصادفی به دو گروه تجربی و شاهد تقسیم شدند. یک روز قبل از شروع تمرینات، آزمون‌های عملکردی شامل تست هاس شارپند رومبرگ (تعادل ایستا با چشم باز و بسته)، تست ستاره (تعادل پویا) و مقیاس برگ (تعادل عملکردی) در هر دو گروه کنترل و تجربی انجام شد. سپس گروه تجربی به انجام یک دوره تمرین ترکیبی هوازی و تعادلی 12 هفته‌ای (چهار جلسه در هفته) پرداختند، در حالی که در این مدت از گروه کنترل خواسته شد که فعالیت‌های روزانه خود را حفظ کنند. پس از اتمام دوره تمرین، پس‌آزمون برای هر دو گروه اجرا شد. داده‌ها به کمک روش‌های آماری t مستقل و تحلیل کواریانس تحلیل شد.  یافته ها اعمال برنامه تمرین ترکیبی هوازی و تعادلی بر بهبود تعادل زنان سالمند تاثیر معنی‌داری داشت (05/0>Р). همچنین نتایج حاکی از تفاوت معنی‌داری بین داده‌های پس‌آزمون، آزمودنی‌های گروه تجربی و گروه شاهد بود (05/0>Р). تفاوت معنی‌داری بین نمرات پیش‌آزمون و پس‌آزمون گروه شاهد مشاهده نشد (05/0<Р). نتیجه گیری براساس یافته‌های این مطالعه، شاید به کارگیری تمرینات ترکیبی هوازی و تعادلی بتواند عاملی تاثیر‌گذار در بهبود تعادل سالمندان باشد.

Title: تبیین عملکرد مراقبتی در سرای سالمندان: یک مطالعه کیفی

http://salmandj.uswr.ac.ir/article-1-763-fa.html

اهداف افزایش جمعیت سالمندان در سال‌های اخیر باعث شده تا میزان بیماری‎های مزمن و در نتیجه نیاز به مراقبت در آن‌ها افزایش چشمگیری پیدا کند. از آنجایی که به دلایل مختلف تعدادی از سالمندان نیاز دارند تا برای مراقبت به سرای سالمندان منتقل شوند، شناسایی عملکرد مراقبتی این مراکز الزامی است و پژوهش حاضر با این هدف انجام گرفته است. مواد و روش ها در این پژوهش که به صورت کیفی انجام شد، 23 مصاحبه نیمه ساختارمند (14سالمند و 7 مراقب و 2 مدیر) در سه سرای سالمندان در شهر اصفهان به عمل آمد. تحلیل داده‌ها با استفاده از روش تحلیل محتوای سنتی انجام شد که جهت تعیین مفاهیم اصلی، مصاحبه‌ها به دقت بررسی و کدگذاری اولیه شد. این کدها چندین بار مورد بازبینی و ارزیابی و طبقه بندی قرار گرفت تا به اشباع اطلاعات منجر شد و در‌نهایت زیر طبقات و طبقات اصلی بدست آمد. آنالیز داده‌ها به طور مداوم و هم‌زمان با جمع‌آوری داده‌ها انجام شد. یافته ها پس از کدگذاری اولیه، تعداد 595 کد اولیه استخراج شد که در نهایت تعداد 9 زیرطبقه و 2 طبقه‌ی اصلی با عناوین مراقبت‌های در‌دسترس و رفع تکلیف حاصل شد. نتیجه گیری مراقبت‌های ارائه شده بیشتر براساس اقدامات روتین و با توجه به جنبه‌های جسمی و رفع نیازهای آنی سالمندان بود، اگرچه به جنبه‌های روانی نیز پرداخته می‌شد. یافته‌های این مطالعه برای مدیران و برنامه‌ریزان مراقبتی در سرای سالمندان به عنوان راهنما جهت در نظر داشتن نیازهای جسمانی و روانی سالمندان کارایی دارد; همچنین بر نیاز به آموزش‌های بالینی جهت پرسنل و تدوین پروتکل‌های درمانی و نظارتی نیز تأکید می‌کند.

Title: مقایسه تاثیر ورزش درمانی و رژیم غذایی با رویکرد کاهش وزن بر سطح افسردگی زنان سالمند با افسردگی شدید

http://salmandj.uswr.ac.ir/article-1-773-fa.html

اهداف تحقیقات اخیر نشان از شیوع بالای افسردگی شدید و چاقی در سالمندان دارند. اما تحقیقی جامع در خصوص اثربخشی کاهش وزن بر میزان افسردگی وجود ندارد. هدف از پژوهش حاضر مقایسه تاثیر ورزش درمانی و رژیم غذایی با رویکرد کاهش وزن بر سطح افسردگی زنان سالمند با افسردگی شدید بود. مواد و روش ها اطلاعات مربوط به افسردگی و ترکیب بدن 1265 زن سالمند چاق (قد: 1/3±7/160سانتی‌متر، سن: 8/6±1/61 سال، وزن: 07/52±1/81 کیلوگرم) در مرکز پزشکی ورزشی کنترل وزن مورد بررسی قرار گرفت. از این میان تعداد 175 آزمودنی که بر اساس پرسشنامه افسردگی بک دارای افسردگی با درجه شدید (8/6±2/30) بودند، انتخاب شدند وبه صورت تصادفی در 3 گروه فعالیت بدنی (59=(n، رژیم غذایی (59=(n و کنترل (57=n) تحت بررسی قرار گرفتند. در مرحله پیش آزمون، تمام متغیرهای چاقی با استفاده از دستگاه تحلیل ترکیب بدن اندازه‌گیری شد. پروتکل تمرینی شامل پنج جلسه تمرین هوازی 45 دقیقه‌ای در هفته به مدت 12 ماه و با شدت حداکثر ضربان قلب 65%-55% بود. برنامه غذایی آزمودنی‌ها بر اساس اطلاعات استخراج شده از بدن آن‌ها و بر اساس میزان کالری توصیه‌شده‌‎ روزانه توسط کارشناس تغذیه تنظیم و ارائه شد. یافته ها برنامه‌های تمرینی و تغذیه‌ای آزمودنی‌ها با استفاده از تلفن، مشاوره حضوری در کلینیک و یا خودگزارش‌دهی انجام می‌شد. مقطع دوم جمع‌آوری داده‌ها پس از گذشت 12 ماه (اتمام پروتکل تمرینی و اجرای رژیم غذایی) انجام شد. پس ازجمع‌آوری داده‌ها، آزمون آنالیز واریانس یک طرفه و آزمون تعقیبی توکی جهت تحلیل داده‌ها مورد استفاده قرار گرفت.  نتایج تحقیق نشان داد که هر دو گروه آزمایشی (تمرین و رژیم غذایی) نسبت به گروه کنترل کاهش بیشتری در سطح افسردگی داشتند (05/0≥P). علاوه بر این، گروه دارای فعالیت بدنی (همراه با کاهش بیشتر چربی احشایی) بهبود بیشتری را در افسردگی نسبت به گروه رژیم غذایی داشت (به ترتیب گروه فعالیت بدنی و رژیم غذایی 001/0P=و 006/0P=). نتیجه گیری نتیجه کلی این است که کاهش وزن چربی می‌تواند باعث کاهش افسردگی شود. اگر این کاهش وزن با تقلیل چربی احشایی ازطریق فعالیت بدنی همراه باشد، نتایج بهتری حاصل خواهد شد.

Title: میزان افسردگی و عوامل مرتبط با آن در سالمندان مقیم سرا: یک مطالعه غربالگری در خرم آباد

http://salmandj.uswr.ac.ir/article-1-761-fa.html

اهداف هدف از مقاله حاضر، بررسی وضعیت افسردگی در میان سالمندان مقیم آسایشگاه صدیق شهر خرم‌آباد و عوامل مرتبط با آن بود. مواد و روش ها این مطالعه یک مطالعه توصیفی-تحلیلی بود. واحد نمونه‌گیری، سالمندان مقیم سرای سالمندان صدیق شهر خرم آباد در محدوده سنی 58 تا 92 سال در سال 1392 بودند که از آن میان سالمندان 60 سال به بالا به روش سرشماری انتخاب شدند. وضعیت افسردگی در آنها با مقیاس 15 سوالی افسردگی سالمندان که برای سالمندان ایران استاندارد شده، مورد ارزیابی قرار گرفت. جهت سنجش ارتباط بین افسردگی و فاکتورهای جمعیت شناختی از آزمون تی مستقل و تست دقیق فیشر و نرم‌افزار SPSS نسخه 19 استفاده شد. یافته ها در این مطالعه، 36% افراد فاقد افسردگی و 64% درجاتی از افسردگی را نشان دادند. از میان افراد افسرده، %2/28 افسردگی خفیف، %2/19 افسردگی متوسط و %6/16 افسردگی شدید داشتند. بین افسردگی سالمندان و سن و سطح تحصیلات آنان رابطه آماری معنی‌داری مشاهده شد. به طوری‌که با افزایش سن، شیوع افسردگی کاهش می‌یافت (000/0>P) و با افزایش سطح تحصیلات شیوع افسردگی کمتر مشاهده می‌شد (01/0>P). بین شیوع افسردگی و سایر متغیرهای جمعیت‌شناختی رابطه آماری معنی‌داری مشاهده نشد. نتیجه گیری با توجه به شیوع بالای افسردگی در جمعیت سالمندان مقیم آسایشگاه‌ها، بررسی و غربالگری سالمندان مقیم سرا به ویژه سالمندان جوان‌تر و دارای سطح تحصیلات پایین‌تر از نظر ابتلای به افسردگی ضروری به نظر می‌رسد.

Title: ارتباط خودکارآمدی عمومی و کیفیت زندگی در سالمندان مقیم سراهای سالمندی استان چهارمحال‌وبختیاری

http://salmandj.uswr.ac.ir/article-1-815-fa.html

اهداف تحلیل رفتن قوای جسمانی و ذهنی سالمندان همراه با بیماری‌های متعدد دوران سالخوردگی، کیفیت زندگی آن‌ها را به شدت تحت تاثیر قرار می‌دهد. با این‌حال خودکارآمدی می‌تواند کیفیت زندگی را بهبود بخشد. لذا مطالعه حاضر با هدف تعیین ارتباط خودکارآمدی عمومی و کیفیت زندگی در سالمندان مقیم سراهای سالمندی انجام شد. مواد و روش ها پژوهش حاضر مقطعی از نوع همبستگی است که در آن 87 نفر سالمند مقیم سراهای سالمندی استان چهارمحال و بختیاری (شهرکرد) به روش سرشماری انتخاب شدند. داده‌ها بوسیله فرم مشخصات فردی، مقیاس خودکارآمدی عمومی (10-GSE) و پرسشنامه کوتاه کیفیت زندگی (36-SF) جمع‌آوری و با روش‌های آماری توصیفی و استنباطی (آزمون تی مستقل، آنالیز واریانس، کروسکال والیس، همبستگی پیرسون و آنالیز رگرسیون) تجزیه و تحلیل شدند. یافته ها براساس نتایج 69% از سالمندان از خودکارآمدی عمومی پائین (02/8±95/22) و 65/5% از کیفیت زندگی پائین (59/18±56/41) برخوردار بودند. در این مطالعه همبستگی مستقیم و معناداری بین خودکارآمدی عمومی و خرده مقیاس‌های عملکرد فیزیکی (701/0=r)، ایفای نقش جسمی (350/0=r)، ایفای نقش عاطفی (354/0=r)، عملکرد اجتماعی (821/0=r)، سلامت روانی (722/0=r)، سرزندگی و نشاط (749/0=r)، درد بدنی (783/0=r)، سلامت عمومی کیفیت زندگی (597/0=r) و همین‌طور کیفیت زندگی کل (797/0=r) مشاهده شد (01/0P< در تمام موارد).  نتیجه گیری کیفیت زندگی ضعیف سالمندان مقیم سراهای سالمندی یک یافته مهم است که نیاز به توجه ویژه دارد. وجود ارتباط مثبت معنادار بین خودکارآمدی عمومی سالمندان و تمام حیطه‌های کیفیت زندگی در این مطالعه، می‌تواند نویدبخش امکان ارتقای کیفیت زندگی این افراد با طراحی و اجرای مداخلات آموزشی و حمایتی جهت تقویت باورهای خودکارآمدی آن‌ها باشد.

Title: تاثیر تمرینات هوازی با شدت کم و متوسط بر کیفیت خواب سالمندان

http://salmandj.uswr.ac.ir/article-1-712-fa.html

اهداف تحقیق حاضر با هدف برررسی تاثیر تمرینات هوازی با شدت کم و متوسط بر کیفیت خواب سالمندان انجام شد. مواد و روش ها آزمودنی‌ها شامل 45 سالمند مرد در دامنه سنی 60 تا 70 سال بودند که به‌طور داوطلبانه انتخاب و به روش تصادفی در دو گروه آزمایش و کنترل گمارده شدند. ابتدا تمامی سالمندان توسط یک پزشک متخصص ارزیابی شدند تا سلامت کامل جسمانی و روانی آن‌ها مورد تایید قرار گیرد. سپس حداکثر ضربان قلب افراد با استفاده از کم کردن سن هر فرد از عدد 220 به دست آمد. در ادامه، بر اساس روش تمرینات هوازی با شدت کم و متوسط، حداکثر ضربان قلب هدف در هر یک از آزمودنی‌های گروه‌های تمرینی محاسبه شد. پروتکل تمرینی افراد شامل 8 هفته (هفته‌ای دو جلسه) تمرین هوازی در قالب آزمون یک مایل پیاده‌روی/دویدن راکپورت بود. شدت تمرین کم تا متوسط نیز با استفاده از بلت‌های مخصوصی که به سینه آزمودنی‌ها متصل شده بود، اندازه‌گیری و کنترل شد. همچنین تمامی آزمودنی‌ها در مراحل پیش‌آزمون (قبل اعمال مداخله) و پس‌آزمون (پس از اعمال مداخله) پرسش‌نامه کیفیت خواب پیتزبرگ را تکمیل کردند. یافته ها نتایج در مرحله پیش‌آزمون نشان داد کیفیت خواب و مولفه‌های آن در سالمندان دو گروه اختلاف معنی‌داری با یکدیگر نداشتند (05/0P>). نتایج تحلیل واریانس یک راهه در مرحله پس‌آزمون کیفیت خواب و مولفه‌های آن نشان داد گروه تمرین با شدت متوسط دارای کیفیت خواب بهتری نسبت به دو گروه دیگر بود (05/0P<). همچنین گروه تمرین با شدت کم نیز در کیفیت خواب و مولفه‌های آن دارای نمرات بهتری نسبت به گروه کنترل بودند (05/0P<).  نتیجه گیری به‌طور کلی تحقیق حاضر نشان داد تمرینات هوازی با شدت متوسط تاثیر مثبتی بر بهبود کیفیت خواب و مولفه‌های آن دارد، لذا انجام تمرینات هوازی با شدت متوسط به عنوان یک روش مفید درمانی به سالمندان و مدیران حوزه سلامت سالمندان جامعه توصیه می‌شود.

Title: تاثیر یک جلسه فعالیت استقامتی برمیزان مایواستاتین پلاسما در مردان سالمند سالم

http://salmandj.uswr.ac.ir/article-1-585-fa.html

اهداف هدف از این تحقیق بررسی تاثیر یک جلسه فعالیت استقامتی بر میزان مایواستاتین به عنوان مهمترین پروتئین درگیر در فرآیند سارکوپنیا در مردان سالمند سالم بود. مواد و روش ها دراین تحقیق 11 مرد سالمند سالم (سن 1/2±68 سال، قد 3/1±177 سانتی‌متر، وزن 5/79±1 کیلوگرم)، به صورت داوطلبانه، مشارکت داشتند. در ابتدا، اندازه‌گیری فاکتورهای توصیفی و فیزیولوژیکی از آزمودنی‌ها به عمل آمد و 72 ساعت پس ازتعیین حداکثر اکسیژن مصرفی (VO2max)، آزمودنی‌ها یک فعالیت استقامتی را با شدت 70 درصد VO2max بر روی دوچرخه کارسنج اجرا نمودند. به منظور بررسی تاثیر فعالیت استقامتی بر میزان پروتئین مایواستاتین پلاسما، سه نمونه خونی، قبل، بلافاصله و چهار ساعت بعد از فعالیت، از ورید بازویی آزمودنی‌ها گرفته شد. میزان مایواستاتین پلاسما با روش الایزا اندازه‌گیری شد. جهت تجزیه و تحلیل آماری داده‌های تحقیق، از آزمون آماری One-way ANOVA استفاده شد. سطح معناداری P≤0/05 در نظر گرفته شد. یافته ها  نتایج این تحقیق نشان داد که بلافاصله و چهار ساعت بعد از فعالیت، میزان مایواستاتین پلاسما به ترتیب %57/54 (P=0/002) و %35/35 (P=0/001) به طور معناداری کاهش یافت. همچنین میزان مایواستاتین پلاسما در چهار ساعت بعد از فعالیت نسبت به بلافاصله بعد ازفعالیت %78/51 کاهش داشت، اما این کاهش معنادار نبود.  نتیجه گیری یافته‌های برآمده از این پژوهش، نشان داد که میزان مایواستاتین پلاسما، در پاسخ به فعالیت استقامتی در مردان سالمند سالم، کاهش معناداری می‌یابد. بنابر یافته‌های این تحقیق احتمال دارد با تجویز فعالیت استقامتی بتوان موجب کاهش میزان مایواستاتین و متعاقباً سارکوپنیا درافراد سالمند شد.

Title: نگاهی بر آثار اقتصادی پدیده سالمندی

http://salmandj.uswr.ac.ir/article-1-702-fa.html

اهداف پیری جمعیت، پدیده‌ای است که در سال‌‌های اخیر نگرانی‌هایی را در کشورهای در حال توسعه به دنبال داشته است. پیش‌بینی‌های انجام گرفته نشان می‌دهد که ایران پس از امارات و بحرین، سومین کشور جهان از نظر سرعت پیری جمعیت بوده است. مطالعه حاضر بر آن است تا مروری بر جنبه‌های اقتصادی سالمندی در مطالعات صورت گرفته در این حوزه داشته باشد. مواد و روش ها برای نیل به این هدف، مقالات موجود در این حوزه گردآوری و با توجه به هدف مطالعه، مقالات مرتبط از میان آنها انتخاب و روش‌شناسی و یافته‌های آن‌ها تلخیص شد. با توجه به جدید بودن موضوع برای اقتصاد ایران، غالب مطالعات استفاده شده مربوط به کشورهای توسعه یافته بوده است. یافته ها نتایج حاصل نشان می‌دهد که حوزه‌های مختلف اقتصاد، اعم از بازار کار، بازار سرمایه‌های فیزیکی و مالی، صندوق‌های بازنشستگی، سرمایه انسانی، مصرف و پس‌انداز، بودجه دولت و مهمتر از همه رشد اقتصادی، به شدت، از کهولت سن در جامعه تاثیر خواهد پذیرفت. نتیجه گیری با توجه به این که تاکنون آثار محدود و بدیهی سالمندی (مانند صندوق‌های بازنشستگی و بودجه دولت) مورد توجه بوده، مطالعه حاضر نگرانی از این پدیده را می‌تواند در حوزه‌های بیشتری برانگیخته و برخورد سیاست‌گذاران هر کدام از این حوزه‌ها را برای مواجهه با آثار سالمندی جدی‌تر نماید.
